# Development trajectory of postural control in stroke patients with hemiplegia: a differential analysis of psycho-social adjustment

**DOI:** 10.3389/fmed.2026.1782383

**Published:** 2026-03-19

**Authors:** Jin Li, Huan He, Yu-hao Wang, Ai-ping Jin

**Affiliations:** 1 School of Medicine, Tongji University, Shanghai, China; 2 Shanghai Tenth People's Hospital, Shanghai, China; 3 Department of Neurology, Henan Provincial People's Hospital, Zhengzhou, China; 4 Department of Neurology, Shanghai Tongren Hospital, Shanghai, China

**Keywords:** curves, hemiplegia, influencing factors, longitudinal studies, postural control, psycho - social adjustment, stroke, trajectory

## Abstract

**Objective:**

To explore the trajectory of postural control and its influencing factors in stroke patients with hemiplegia, and to explore the correlation between different trajectories and psycho-social adjustment.

**Methods:**

A cohort of 225 stroke patients with hemiplegia was assessed using the Postural Assessment Scale for Stroke Patients (PASS) and the Self-Report Psycho-social Adjustment to Illness Scale (PAIS-SR) at five time points from discharge to 6 months post-discharge (T1-T5), with data analyzed via latent class growth modeling, factor analysis, and ANOVA.

**Results:**

Three postural control trajectories were identified: C1 significant improvement group, C2 slow improvement group, and C3 no improvement group. There were significant differences in age (*χ*^2^ = 15.486, *p =* 0.004), self - rated anxiety and depression (*χ*^2^ = 7.791, *p =* 0.020), social support (*χ*^2^ = 9.644, *p =* 0.047), psychological distress (*χ*^2^ = 8.110, *p =* 0.017), and coping strategies (*χ*^2^ = 7.545, *p =* 0.017) among different trajectory categories. Logistic regression analysis showed that age, anxiety and depression, social support, psychological distress, and coping strategies were the influencing factors of the trajectory of postural control in stroke patients with hemiplegia (*p <* 0.05). After stroke onset (T1 - T5), the scores of PAIS-SR in each group showed a downward trend (indicating improved adjustment, as higher adjustment corresponds to lower PAIS-SR scores) [C1: (*F* = 170.335, *p <* 0.001); C2: (*F* = 5.614, *p =* 0.005); C3: (*F* = 1.993, *p =* 0.186)]. At the T1 stage, there was no significant difference among the three groups. At the T2 - T3 stage, the PAIS-SR score of group C1 was significantly lower than that of group C2 and group C3 (*p <* 0.01). At the T4 - T5 stage, the PAIS-SR score of group C1 was significantly lower than that of group C2 and group C3 (*p <* 0.001), and the PAIS-SR score of group C2 was significantly lower than that of group C3 (*p <* 0.001).

**Conclusion:**

For stroke patients with hemiplegia, an individualized rehabilitation program should be implemented, along with early intervention of postural control training and combined with psychosocial support intervention.

## Introduction

According to the latest data from the Global Stroke Report 2025, there were 93.816 million stroke cases worldwide in 2024, and the age - standardized prevalence was 1,099 per 100,000. There were 11.946 million new stroke cases, and the age - standardized incidence rate was 142 per 100,000 (1). Among them, 65.3% (7.804 million) were ischemic stroke, 28.8% (3.444 million) were intracerebral hemorrhage, and 5.8% (697,000) were subarachnoid hemorrhage. Globally, stroke caused 7.253 million deaths, accounting for 10.7% of the total global deaths. There were more than 160 million disability - adjusted life years (DALYs), and stroke ranked third in the total burden of global diseases (2, 3).

China is the country with the highest burden of stroke in the world. In 2024, the age - standardized prevalence of stroke was 1301.4 per 100,000, the age - standardized incidence was 204.8 per 100,000, and the number of new stroke cases reached 4.04 million, accounting for 34.2% of the total number of new cases in the world, far exceeding the proportion of China’s population in the global population (about 20%) (4). Among them, ischemic stroke accounted for 67.8% (2.772 million cases) and cerebral hemorrhage accounted for 28.7% (1.173 million cases) (5).

Postural control refers to the ability of the human body to integrate multi - sensory information such as vision, vestibular sense, and proprioception through the central nervous system and coordinate the musculoskeletal system to maintain the stability and orientation of the body in space (6). Neurological recovery after stroke exhibits significant heterogeneity, with substantial variation in the trajectories and outcomes of motor, sensory, and cognitive function among different patients. This variability in recovery profoundly influences the rehabilitation process of a patient’s postural control. As the foundation for maintaining bodily stability and orientation, postural control relies on multisensory integration and neuromuscular coordination, serving as a critical determinant of functional independence and quality of life after stroke (7). Research indicates that compensatory training and consolidation of postural control throughout the acute, recovery, and maintenance phases post-stroke are beneficial in helping patients adapt to long-term life demands and preventing falls and recurrent strokes (8–13). A dynamic interaction exists between the functional recovery status after stroke and postural control function: the recovery process shapes the path of postural control reconstruction, while the level of postural control, in turn, influences the effectiveness of overall functional recovery and the capacity for participation in daily life. In this context, systematically examining the developmental trajectory of postural control in stroke patients and identifying the factors influencing different recovery patterns is of significant clinical and rehabilitative importance. It is crucial for the early prediction of rehabilitation outcomes, the implementation of stratified interventions, and the optimization of long-term functional results.

Disease psycho - social adjustment refers to the process by which individuals coordinate and adapt to the societal, family, and collective environment by adjusting their cognitive attitude, emotional response, and self - evaluation in the face of disease. It encompasses multiple dimensions, including disease cognition, emotion management, and social role adjustment (14, 15). For stroke patients, psycho - social adjustment holds great significance. Stroke patients often suffer from psychological problems such as anxiety, depression, and low self - esteem due to limb dysfunction, language impairment, and decreased living ability. The incidence of psychological disorders is as high as 20–79% (16, 17). Good psycho - social adjustment can help patients correctly face the reality of the disease, improve negative emotions, and actively cooperate with treatment and rehabilitation training. As a result, it can enhance the speed of neurological function recovery and the quality of life. Studies have shown that patients with a higher level of psycho - social adjustment have significantly lower degrees of fatigue and loneliness, better recovery effects, and a lower risk of recurrence (18, 19). Therefore, in the process of stroke rehabilitation, psycho - social adjustment is not only an important part of disease treatment but also a key factor affecting the overall rehabilitation effect of patients (20).

In view of this, this study used the latent class growth model (LCGM) to identify the trajectory of postural control development in stroke patients with hemiplegia, analyze the influencing factors of different trajectories, and explore their relationship with psycho - social adjustment. This study provides a theoretical basis for the clinical identification of high - risk groups for psycho - social adjustment, the improvement of their psychological stress, and the promotion of prognosis development.

### Study subjects

From January 2024 to December 2024, convenience sampling was employed to select 225 stroke patients with hemiplegia admitted to Shanghai Tenth People’s Hospital/Shanghai Tongren Hospital/Henan Provincial People’s Hospital as the research subjects.

*Inclusion criteria:*

1. Stroke was diagnosed through clinical examinations such as brain CT and MRI.
2. Unilateral onset, with a Rankin scale grade of 3–4.
3. No physical disability prior to the stroke.
4. Adults aged 18 years or older.
5. Possessing basic understanding and communication skills, and all having signed the informed consent.

*Exclusion criteria:*

1. A previous history of mental illness.
2. Receiving medication, psychotherapy, etc.
3. Death or recurrence of stroke during the follow - up.
4. The questionnaire was missing more than once.

Prior to the study initiation, a written application was submitted to and approved by the Ethics Committee of Tongji University (Approval no. 202401-225), as well as the Ethics Committees of the Shanghai Tenth People’s Hospital [no. (2024) 05301], Henan Provincial People’s Hospital (no. HPPH202402361) and Shanghai Tongren Hospital (no. Tongren2024012-05) for clinical investigation.

### Survey tools

#### Questionnaire for basic information

The patients’ demographic data (gender, age, marital status, residence, education level, family monthly income per capita, smoking history, drinking history), disease data (previous comorbidities).

#### Hospital anxiety and depression scale (HADS)

Using the Chinese version HADS (21), it consists of 14 items, with each item scored on a 4-point scale from 0 to 3. The total score ranges from 0 to 21, where 0–7 indicates no anxiety or depression, and ≥8 suggests the presence of anxiety or depression. Studies on its application in internal medicine inpatients in general hospitals have shown that the scale has good reliability and validity, with a Cronbach’s *α* coefficient of 0.932 and test–retest reliability ranging from 0.820 to 0.906.

#### Perceived social support scale (PSSS)

Using the Chinese version PSSS (22), it consists of 12 items divided into three dimensions: family support, friend support, and other support. The scale adopts a Likert 7-point scoring method (1–7 points), with a total score ranging from 12 to 84. The evaluation criteria are as follows: 12–36 indicates low support, 37–60 indicates medium support, and 61–84 indicates high support. In terms of reliability and validity, when applied to inpatients, the total scale’s Cronbach’s *α* coefficient is 0.840, and the coefficients for each dimension range from 0.818 to 0.820.

#### Distress thermometer (DT)

Used to assess the level of psychological distress in patients over the past week (23). The DT consists of only one item, scored from 0 to 10, ranging from “no distress” to “extreme distress,” with higher scores indicating greater psychological distress. In the Chinese inpatient population, when the cutoff score is set at 4, the sensitivity and specificity are 0.88 and 0.70, respectively. In this study, a score of ≥4 was defined as the presence of psychological distress. The Cronbach’s *α* coefficients for the two measurement time points in this study were 0.810 and 0.853.

#### Simplified coping style questionnaire (SCSQ)

Developed by Xie et al. (24), it consists of 20 items divided into two dimensions: positive coping (12 items) and negative coping (8 items). The scale uses a 4-point scoring method from 0 to 3 (0 = not adopted, 3 = frequently adopted), with a total score ranging from 0 to 60. Evaluation typically involves calculating the mean scores of the two dimensions or using the formula “coping tendency = standardized positive coping score - standardized negative coping score” to determine whether an individual primarily adopts a positive (>0) or negative ( < 0) coping style. The scale has good reliability and validity, with a total Cronbach’s *α* coefficient of 0.90, and α coefficients of 0.89 and 0.78 for the positive and negative subscales, respectively.

#### Postural assessment scale for stroke patient (PASS)

The scale is divided into two main parts. One is posture maintenance, which includes five aspects such as sitting and standing without support and standing with support. The second is posture change, which includes seven aspects such as turning over from the supine position to the paralyzed side and moving from lying flat to the sitting position. Each item is scored from 0 to 3, with a total score of 36. A higher score indicates better postural control (25).

#### Self-report psycho-social adjustment to illness scale (PAIS-SR)

The scale was revised by Derogatis (26) and translated into Chinese by Yao (27). It encompasses seven dimensions: healthcare (7 items), work ability (6 items), family relationship (7 items), sexual ability (6 items), communication (5 items), entertainment (6 items), and psychological status (7 items), totaling 44 items. Each item is scored using the Likert 4-level scoring method, with scores ranging from 0 to 3 points per item. “Bad” is scored as 0 points, “good” as 3 points, and the total score ranges from 0 to 132 points. The higher the score, the more severe the patient’s psycho-social adjustment problem. The Cronbach’s *α* coefficient of the scale is 0.872, and the Cronbach’s α coefficient of the scale in this study is 0.880.

### Questionnaire recovery and quality control

This study was conducted after obtaining approval from the hospital ethics committee and informed consent from the patients. A longitudinal survey design was adopted, and data were collected at five time points: before discharge (T1), and at 1, 2, 3, and 6 months after discharge (T2 - T5). PASS was evaluated by the investigators, and PAIS - SR was self - assessed by the participating hemiplegic stroke patients. The T1 questionnaire was completed before discharge, and the T2 - T5 questionnaires were completed during the patients’ out - of - hospital follow - up. All questionnaires were processed anonymously. Data collection and entry were carried out by two researchers in a division of labor: one was responsible for collection, and the other independently checked to ensure accuracy. To encourage participation, patients who completed all questionnaires received a small gift.

### Statistical methods

SPSS 26.0 software and Mplus 8.0 software were used for statistical analysis and data testing. Count data were expressed as cases and percentages (%), and chi - square analysis was employed. Measurement data were presented as mean ± standard deviation (^−^X ± S), and analysis of variance was utilized for data statistics. For the first time, the intra - class variance was set to 0. Starting from one model, the number of models was incremented one by one, and the optimal model was determined based on practical significance and fitting indices. The fitting indices included the Akaike information criterion (AIC), the Bayesian information criterion (BIC), and the sample - corrected BIC (aBIC). Smaller statistical values indicated better model fitting. Entropy represents the classification accuracy, and a value closer to 1 indicates higher accuracy. The likelihood ratio test (LRT) and the bootstrap - based likelihood ratio test (BLRT) were used to compare the fit differences between k - 1 and k - class models. The test level was set as *α* = 0.05. After determining the optimal trajectory model, patients were assigned to their respective trajectory categories. A multinomial logistic regression analysis was conducted, with one category set as the reference, to explore the effects of various influencing factors on trajectory category membership. The results are presented as odds ratios (OR) with their 95% confidence intervals (CI). Repeated-measures analysis of variance (ANOVA) was used to compare differences in psychosocial adaptation scores across the five time points (T1–T5) among patients in different trajectory categories, with post-hoc pairwise comparisons performed using the Bonferroni method.

## Results

### General demographic data

During the follow - up period, 31 patients were lost to follow - up because of stroke recurrence, accidental death, refusal to answer questions, and other reasons. A total of 194 valid questionnaires were collected in this study, with 110 males (56.70%) and 84 females (43.30%). The mean age was (63.22 ± 6.80) years (range: 37–82 years). For details, see Table 1.

**Table 1:** General information of the respondents (*n =* 194).

Items	Categories	*n*	Percentage (%)
Age (years)	< 45	21	10.82
	45–59	67	34.54
	≥60	106	54.64
Gender	Male	110	56.70
	Female	84	43.30
Careers	Enterprises and institutions	19	9.79
	Farmer	55	28.35
	Staff/staff	52	26.80
	Retirement	51	26.29
	No regular occupation	17	8.77
Marital status	Married	174	89.69
	Unmarried	8	4.12
	Divorced/widowed	12	6.19
Place of residence	Rural	72	37.11
	Towns	122	62.89
Monthly income (yuan)	< 3,000	39	20.10
	3,000 -	57	29.38
	5,001 -	72	37.11
	> 8,000	26	13.41
Level of education	Primary school and below	54	27.84
	Junior high school	65	33.51
	High school	42	21.65
	College and above	33	17.00
Comorbid conditions	Hypertension	51	26.29
	Diabetes	44	22.68
	Coronary heart disease	46	23.71
Smoking history	Yes	85	43.81
	No	109	56.19
History of drinking	Yes	79	40.72
	No	115	59.28

Postural control scores of stroke hemiplegic patients at T1-T5 (Table 2).

**Table 2:** Postural control scores of stroke patients with hemiplegia.

Time points	PASS score	PAIS-SR score
T1	17.46 ± 3.21	66.34 ± 5.45
T2	18.51 ± 3.18	65.45 ± 6.56
T3	21.01 ± 3.24	63.87 ± 5.44
T4	23.07 ± 2.61	60.68 ± 5.37
T5	23.61 ± 3.36	59.33 ± 5.53
F	75.667	45.338
P	< 0.001	< 0.001

### Determination of postural control trajectory in stroke patients with hemiplegia

Based on latent class growth analysis, this study constructed models with 1 to 5 potential developmental trajectories. The model fit results of the latent profile analysis are presented in Table 3. For the 1-class model, the latent variables failed to explain the associations among the observed variables, resulting in the poorest model fit. The 3-class model demonstrated a high entropy value of 0.885, and the sample size of each profile exceeded 5% of the total sample, indicating good interpretability. In comparison, the 2-class model showed a lower entropy value of 0.848, and its AIC, BIC, and aBIC values were all higher than those of the 3-class model (where smaller AIC, BIC, and aBIC values indicate better fit). For the 4-class model, the LRT result was not statistically significant (*p =* 0.077), and one of the profiles had a sample size <5% of the total, resulting in poor interpretability. Similarly, the 5-class model also showed a non-significant LRT result (*p =* 0.149). In summary, the 3-class model was selected as the optimal model.

**Table 3:** Comparison of analysis indicators of postural control development trajectory types in stroke patients with hemiplegia (*n =* 194).

Models	AIC	BIC	aBIC	LRT	BLRT	Entropy	Class probability (%)
1	23802.723	24055.783	24007.753				1
2	23185.344	23563.496	23325.945	0.000	0.000	0.848	0.38/0.62
3	22581.574	22942.581	22831.651	0.003	0.000	0.885	0.33/0.41/0.26
4	21674.995	21847.878	21793.493	0.077	0.000	0.887	0.04/0.32/0.35/0.29
5	20263.150	20526.357	20491.405	0.149	0.000	0.902	0.17/0.23/0.11/0.22/0.27

### Average attribution rate and naming of three categories of postural control trajectory

According to the LCGM model and combined with the characteristics of postural control trajectories in stroke patients with hemiplegia, postural control was divided into three subgroups. The average probabilities of stroke patients belonging to each latent category were 0.980, 0.972, and 0.982, respectively (see Table 4).

**Table 4:** Average attribution rate of three types of postural control trajectories (*n =* 194).

Model	C1	C2	C3
C1	0.980	0.014	0.006
C2	0.016	0.972	0.012
C3	0.004	0.014	0.982

Group C1 included 64 patients, accounting for 32.99% of the total. After the disease, the postural control of patients increased significantly from the middle level. So, group C1 was named the “significantly improved postural control group”.

Group C2 included 80 cases, accounting for 41.24% of the total. The postural control of patients gradually increased from the middle level. Thus, it was named the “slowly improved postural control group”.

**Figure 1:**
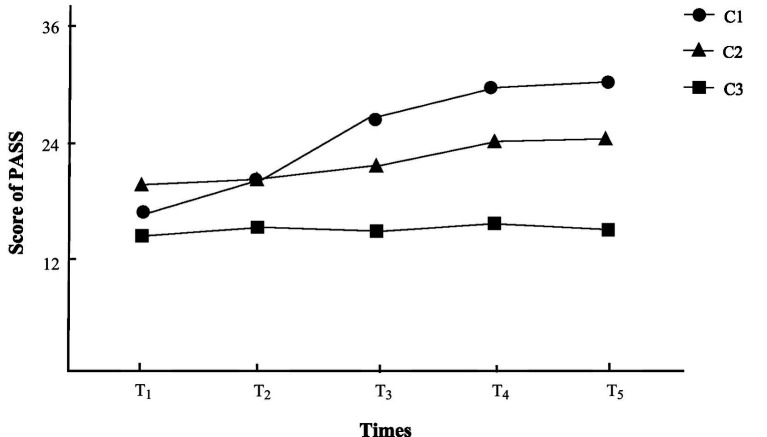
Trajectory of the latent class growth model for postural control in stroke hemiplegic patients.

Group C3 included 50 cases, accounting for 25.77% of the total. The postural control of patients fluctuated around a medium - and - low level. Therefore, group C3 was named the “unimproved postural control group”, and the specific trend is shown in Figure 1.

### Influencing factors of postural control trajectory in stroke patients with hemiplegia

The general data of three latent categories of stroke patients after hemiplegia are compared, and the results show that age (*χ*^2^ = 15.486, *p =* 0.004), self - rated anxiety and depression (*χ*^2^ = 7.791, *p =* 0.020), social support (*χ*^2^ = 9.644, *p =* 0.047), psychological distress (*χ*^2^ = 8.110, *p =* 0.017), and coping strategies (*χ*^2^ = 7.545, *p =* 0.020; *p =* 0.023), as shown in Table 5, had statistically significant differences in the trajectory of postural control in stroke patients with hemiplegia.

**Table 5:** Results of influencing factors analysis of three latent categories of postural control in stroke patients with hemiplegia.

Variables	Grouping	Group C1 (*n =* 64)	Group C2 (*n =* 80)	Group C3 (*n =* 50)	*χ* ^2^	*p*-value
Age (years)	<45	11	8	2	15.486	0.004
	45–59	27	30	10		
	≥60	26	42	38		
Gender	Male	31	44	35	5.476	0.065
	Female	33	36	15		
Marital status	Married	58	70	46	0.994	0.911
	unmarried	2	4	2		
	Divorced/widowed	4	6	2		
Place of residence	Rural	24	29	19	0.046	0.977
	Towns	40	51	31		
Level of education	Primary school and below	19	24	11	2.623	0.854
	Junior high	21	26	18		
	High school	12	16	14		
	Junior college and above	12	14	7		
Income level (yuan)	< 3,000	14	16	9	3.048	0.803
	3,000–5,000	20	26	11		
	5,001–8,000	23	28	21		
	> 8,000	7	10	9		
Occupation	Enterprises/institutions	7	8	4	3.135	0.926
	Farmers	16	22	17		
	Staff/clerks	20	21	11		
	Retirement	16	23	12		
	No occupation	5	6	6		
Comorbid conditions						
Hypertension	Yes	16	22	13	0.118	0.943
	No	48	58	37		
Diabetes	Yes	13	19	12	0.306	0.858
	No	51	61	38		
Coronary heart disease	Yes	15	18	13	0.212	0.899
	No	49	62	37		
Smoking history	Yes	26	36	23	0.407	0.816
	No	38	44	27		
Drinking history	Yes	24	31	24	1.501	0.472
	No	40	49	26		
Anxiety and depression	Yes	44	41	22	7.791	0.020
	No	20	39	28		
Social support	Low support	23	18	9	9.644	0.047
	Medium support	31	44	23		
	High support	10	18	18		
Psychological distress	Yes	18	35	27	8.110	0.017
	No	46	45	23		
Coping strategies	Positive coping	14	25	23	7.545	0.023
	Negative coping	50	55	27		

### Logistic factor analysis of postural control trajectory in stroke patients with hemiplegia

In the multinomial logistic regression model, the group with continuously improved postural control (C1 group) was used as the reference. The independent variables were coded as follows: age as an ordinal variable ( < 45 years = 1, 45–59 years = 2, ≥60 years = 3, with “ < 45 years” as the reference); self-rated anxiety/depression (yes = 1, no = 0, with “no” as the reference) and self-rated psychological distress (present = 1, absent = 0, with “absent” as the reference) as binary variables; self-rated social support as an ordinal variable (low = 1, medium = 2, high = 3, with “low” as the reference); and coping style as a binary variable (active coping = 1, passive coping = 2, with “passive coping” as the reference).

Logistic regression analysis showed that age, anxiety/depression, social support, psychological distress, and coping style were factors influencing the trajectories of postural control changes in stroke patients with hemiplegia (*p <* 0.05). Specifically:

When compared with the continuously improved group (C1 group), the unimproved group (C3 group) showed the following results.

Compared with patients aged < 45 years, those aged 45–59 years (OR = 1.383, 95% CI: 1.065–1.794, *p =* 0.020) and ≥60 years (OR = 1.534, 95% CI: 1.166–2.019, *p =* 0.001) had a significantly higher risk of belonging to the C3 group. Patients with self-rated anxiety/depression had 1.699 times the risk of belonging to the C3 group compared with those without anxiety/depression (OR = 1.699, 95% CI: 1.264–2.284, *p <* 0.001). Compared with patients reporting low social support, those with medium (OR = 0.709, 95% CI: 0.585–0.859, *p <* 0.001) or high social support (OR = 0.587, 95% CI: 0.462–0.746, *p <* 0.001) had a significantly lower risk of belonging to the C3 group. Patients with self-rated psychological distress had 1.539 times the risk of belonging to the C3 group compared with those without distress (OR = 1.539, 95% CI: 1.154–2.053, *p =* 0.004). Compared with patients using passive coping styles, those using active coping styles had a significantly lower risk of belonging to the C3 group (OR = 0.567, 95% CI: 0.432–0.743, *p <* 0.001).

When compared with the continuously improved group (C1 group), the slowly improved group (C2 group) showed the following results.

The directions of the effects of all influencing factors were completely consistent with those in the “C1 vs. C3” comparison, and all were statistically significant. Specifically, older age, presence of anxiety/depression, or psychological distress were risk factors for belonging to the C2 group, while higher levels of social support and active coping styles were protective factors (all *p <* 0.05). Details are presented in Table 6.

**Table 6:** Logistic factor analysis of postural control trajectories in stroke patients with hemiplegia (*n =* 194).

Items	B	SD	Walds *χ*^2^	*P*	OR	95% CI
C3 vs. C1						
Constant	2.011	0.497	16.372	< 0.001	–	–
Age (years)						
45–59	0.324	0.133	5.935	0.020	1.383	1.065–1.794
≥60	0.428	0.140	9.346	0.001	1.534	1.166–2.019
Anxiety and depression						
Yes	0.530	0.151	12.320	< 0.001	1.699	1.264–2.284
Social support						
Medium support	−0.344	0.098	12.322	< 0.001	0.709	0.585–0.859
High support	−0.532	0.122	19.015	< 0.001	0.587	0.462–0.746
Psychological distress						
Yes	0.431	0.147	8.596	0.004	1.539	1.154–2.053
Coping strategies						
Positive coping	−0.568	0.138	16.941	< 0.001	0.567	0.432–0.743
C2 vs. C1						
Constants	1.695	0.345	24.138	< 0.001	–	–
Age (years)						
45–59	0.301	0.112	7.223	0.012	1.351	1.085–1.683
≥60	0.395	0.164	5.801	0.046	1.484	1.076–2.047
Anxiety and depression						
Yes	0.412	0.089	21.430	< 0.001	1.510	1.268–1.798
Social support						
Medium support	−0.292	0.091	10.296	< 0.001	0.747	0.625–0.893
High support	−0.430	0.095	20.488	< 0.001	0.651	0.540–0.784
Psychological distress						
Yes	0.387	0.135	8.218	0.006	1.473	1.130–1.919
Coping strategies						
Positive coping	−0.472	0.113	17.447	< 0.001	0.624	0.500–0.778

### Differences in psycho - social adjustment among stroke patients with hemiplegia having different postural control trajectories

A comparative analysis of the psycho - social adjustment of patients with cerebral apoplexy and hemiplegia with different postural control trajectories after illness was conducted. The results showed that after cerebral apoplexy and hemiplegia (from T1 to T5), the psycho - social adjustment scores of patients in each group presented a downward trend [C1: (*F* = 170.335, *p <* 0.001); C2: (*F* = 5.614, *p =* 0.005); C3: (*F* = 1.993, *p =* 0.186)].

At T1, there was no significant difference among the three groups. At the T2 - T3 stage, the psycho - social adjustment score of group C1 was significantly lower than those of group C2 and group C3 (*p <* 0.01). At the T4 - T5 stage, the psycho - social adjustment score of group C1 was significantly lower than those of group C2 and group C3 (*p <* 0.001), and the psycho - social adjustment score of group C2 was significantly lower than that of group C3 (*p <* 0.001). See Table 7 for details.

**Table 7:** Analysis of differences in psycho-social adjustment of stroke patients with different postural control trajectories.

Variables	Psycho-social adjustment score					F	*P*
	T1	T2	T3	T4	T5		
C1	66.15 ± 7.58	64.28 ± 6.67	61.15 ± 6.16	55.38 ± 6.61	52.94 ± 5.76	170.335	< 0.001
C2	66.23 ± 7.61	65.95 ± 7.57	64.99 ± 6.72	62.17 ± 6.02	61.15 ± 6.97	5.614	0.005
C3	66.74 ± 6.54	66.13 ± 6.60	65.56 ± 6.51	65.08 ± 5.40	64.59 ± 6.56	1.993	0.186
F	0.135	4.585	6.342	16.594	24.372	–	–
P	0.834	0.008	0.001	< 0.001	< 0.001	–	–
Comparing the two results	–	C1 < C2^***^, C1 < C3^**^	C1 < C2^***^, C1 < C3^**^	C1 < C2^***^, C1 < C3^***^, C2 < C3^***^	C1 < C2^***^, C1 < C3^***^, C2 < C3^***^	–	–

**P < 0.01, ***P < 0.001.

## Discussion

### The postural control of hemiplegic stroke patients demonstrated three distinct trajectories

The growth mixture model identified three types of postural control trajectories after stroke - induced hemiplegia: the significant improvement in postural control group, the slow improvement in postural control group, and the no improvement in postural control group. There are notable individual differences in the recovery process following a stroke, and not all patients can achieve the same recovery outcome. This implies that individualized rehabilitation strategies should be employed in clinical practice.

Secondly, the categorization into the significant postural control improvement group, slow postural control improvement group, and no postural control improvement group reflects the comprehensive impact of factors such as neuroplasticity, underlying medical conditions, and rehabilitation compliance in different patients (28, 29). Differences in neuroplasticity likely serve as the central mechanism underlying the divergent trajectories of postural control recovery observed in patients. Factors such as age, the intensity and timing of rehabilitation, as well as the extent of the initial brain injury, collectively shape an individual’s latent and efficiency for neuroplastic change. By identifying and targeting these patient-specific modulators of neuroplasticity, clinicians can develop more personalized rehabilitation strategies, offering a critical pathway toward optimizing functional recovery. Postural control is the foundation of motor function, and its recovery level directly determines the patient’s walking ability, balance function, and capacity for activities of daily living. Patients in the non - improvement group confront higher risks of falls and functional dependence (30, 31).

### Postural control of stroke patients with hemiplegia is affected by many factors

This study found that age, anxiety and depression, social support, psychological distress, and coping strategies are important factors affecting the trajectory of postural control in stroke patients with hemiplegia. These factors act on the recovery of neurological function and the rehabilitation process through multiple mechanisms.

Age factors mainly affect neuroplasticity and physical function reserve. In elderly patients, the brain’s ability to self - reorganize and learn new skills is relatively weak. At the same time, physiological functions such as muscle strength, balance, and coordination naturally decline with age, which directly limits the recovery latent of postural control (32, 33).

Anxiety and depression affect the recovery latent of postural control through neurobiochemical changes and psychological stress. The incidence of post - stroke depression is as high as 20–60%. Its pathogenesis is related to the damage of brain tissue, leading to the damage of norepinephrine and serotonergic neurons and their conduction pathways. The dysfunction of these neurotransmitters not only affects emotional regulation but also reduces initiative and compliance (34, 35). Anxiety and depression can also cause a series of physiological reactions such as muscle tension, increased blood pressure, and sleep disorders, which directly interfere with the process of neuroplasticity, i.e., the brain’s ability to reorganize itself and learn new skills, and thus hinder the recovery of postural control function.

As an important external resource, social support can significantly improve the mental state of patients, enhance rehabilitation confidence, and improve treatment compliance through multi - dimensional effects such as emotional support, information support, and material support (36, 37). A good social support network can reduce the psychological pressure of patients and promote their physical self - healing ability. At the same time, by providing correct rehabilitation guidance and continuous encouragement, it can help patients establish a positive attitude toward rehabilitation, thereby playing a positive regulatory role in the rehabilitation process (38).

For disease patients, psychological distress, which reflects the subjective experience and the difficulty in dealing with the disease, can lead to excessive mental pain, negative emotions, self - denial, and even the idea of giving up treatment in patients. This negative psychological state will affect the immune function through the neuroendocrine system and reduce the effect (39) of rehabilitation training.

Coping strategies are psychological strategies for individuals to face disease and rehabilitation challenges. Positive coping strategies such as actively seeking help and maintaining an optimistic attitude can promote the recovery of neurological function, while negative coping strategies such as avoidance and denial can delay the recovery process (40, 41).

These psychological factors jointly shape the change trajectory of postural control function in stroke patients with hemiplegia by affecting rehabilitation motivation, training participation, and neuroplasticity.

### The relationship between different trajectories of postural control and psycho - social adjustment in stroke patients with hemiplegia

The study results showed that the psycho - social adjustment scores of stroke patients with hemiplegia exhibited a declining trend over time, indicating a gradual improvement in psycho - social adjustment levels as rehabilitation progressed. This improvement is primarily attributed to the patients’ psychological adaptation process to the disease.

At the T1 stage, patients in all three groups were in a state of shock and denial regarding the disease, lacking psychological preparation for the sudden onset and exhibiting fear, anxiety, and emotional numbness. At this time, the level of psycho-social adjustment was low (reflected by higher scores), with no significant differences between groups.

As treatment and rehabilitation progressed (T2 to T3 stages), patients in the C1 group gradually recognized the controllability of the disease and the possibility of recovery through active rehabilitation training and psychological interventions, transitioning from the denial phase to the adaptation phase. Their psychological state improved significantly, resulting in a notably higher level of psycho-social adjustment compared to the C2 and C3 groups.

By the T4 to T5 stages, C1 group patients had largely adapted to the disease state, actively cooperating with treatment and engaging in rehabilitation training, achieving a high level of psycho-social adjustment. Although the C2 group showed some improvement, their rehabilitation outcomes and psychological adaptation were not as pronounced as those of the C1 group. Meanwhile, the C3 group, possibly due to a lack of systematic rehabilitation or psychological support, remained in a state of depression or anxiety, exhibiting the lowest level of psycho-social adjustment.

The absence of statistically significant differences between groups suggests that all patients underwent a psychological adjustment process from denial to adaptation, albeit to varying degrees of improvement.

Based on the above findings, the psychosocial adaptation of stroke patients with hemiplegia is a dynamic evolutionary process, and the degree of improvement varies significantly among groups with different recovery trajectories. This conclusion points to a clear direction for clinical practice: a shift from a homogeneous management model to precise, stage-specific, and differentiated intervention strategies. The core insight is that psychological rehabilitation should no longer be viewed as generalized support but rather constructed as a precision intervention system capable of synchronizing with the patient’s physiological recovery stage, psychological adaptation process, and individual recovery potential.

In the post-acute phase, the primary goal is to establish a sense of safety, utilizing stabilization techniques to alleviate traumatic stress while avoiding premature attempts to alter cognition. During the rehabilitation phase, the focus should shift toward cognitive-behavioral restructuring: for patients with significant recovery, reinforce positive beliefs and decompose goals; for those with slow improvement, use behavioral activation to break avoidance cycles. In the community integration stage, the emphasis is on rebuilding a meaningful life: well-adapted patients should leverage their strengths and peer support, while those still facing difficulties require family interventions and integrated community resources to enhance adaptation across multiple levels.

### Limitations

This study also has certain limitations. The use of a convenience sampling method may limit the generalizability of the findings. Furthermore, the assessment of social adaptation ability relied on patient-reported outcomes, which may increase the risk of bias. To address these issues, future research will focus on conducting multi-center studies and employing more objective measurement tools to refine the results. Finally, the patient cohort in this study included both hemorrhagic and ischemic stroke cases, and as their prognoses and postural control may differ, subsequent studies will evaluate and analyze the two groups separately to further optimize the research findings.

## Conclusion

There are three dynamic trajectories of postural control in stroke patients with hemiplegia within 6 months after onset. Age, anxiety and depression, social support, psychological distress, and coping strategies are the independent factors affecting the trajectory differentiation. The different developmental trajectories of postural control are closely related to the psycho - social adjustment of stroke patients.

For stroke patients with hemiplegia, individualized rehabilitation programs should be implemented, including early intervention of postural control training combined with psychosocial support intervention. According to the influencing factors such as patients’ age, anxiety and depression, social support level, psychological distress, and coping strategies, a staged and multi - dimensional rehabilitation plan should be formulated to promote the collaborative recovery of postural control and psycho - social adjustment, and improve the overall rehabilitation effect and quality of life of patients.

## Data Availability

The original contributions presented in the study are included in the article/supplementary material, further inquiries can be directed to the corresponding author.
